# To Beg, or Not to Beg? That Is the Question: Mangabeys Modify Their Production of Requesting Gestures in Response to Human’s Attentional States

**DOI:** 10.1371/journal.pone.0041197

**Published:** 2012-07-18

**Authors:** Audrey Maille, Lucie Engelhart, Marie Bourjade, Catherine Blois-Heulin

**Affiliations:** 1 UMR 6552 Ethos «Ethologie Animale et Humaine», University of Rennes 1 - CNRS, Station biologique, Paimpont, France; 2 Research Center in the Psychology of Cognition, Language and Emotion, Aix-Marseille University, Aix-en-Provence, France; Weill Cornell Medical College, United States of America

## Abstract

**Background:**

Although gestural communication is widespread in primates, few studies focused on the cognitive processes underlying gestures produced by monkeys.

**Methodology/Principal Findings:**

The present study asked whether red-capped mangabeys (*Cercocebus torquatus*) trained to produce visually based requesting gestures modify their gestural behavior in response to human’s attentional states. The experimenter held a food item and displayed five different attentional states that differed on the basis of body, head and gaze orientation; mangabeys had to request food by extending an arm toward the food item (begging gesture). Mangabeys were sensitive, at least to some extent, to the human’s attentional state. They reacted to some postural cues of a human recipient: they gestured more and faster when both the body and the head of the experimenter were oriented toward them than when they were oriented away. However, they did not seem to use gaze cues to recognize an attentive human: monkeys begged at similar levels regardless of the experimenter’s eyes state.

**Conclusions/Significance:**

These results indicate that mangabeys lowered their production of begging gestures when these could not be perceived by the human who had to respond to it. This finding provides important evidence that acquired begging gestures of monkeys might be used intentionally.

## Introduction

In primates, gestural communication involves manual and bodily gestures which can be clustered into visual, auditory or tactile signals, depending on the perceptual system used by the recipient to perceive them [1]. Gestures are used to communicate with conspecifics in intimate social contexts such as play, grooming, nursing or agonistic encounters (for a review, see [2]). Moreover, gestures can be exhibited by captive nonhuman primates interacting with humans, to request distant objects. Yet, a gulf seems to divide monkeys from apes in their production of spontaneous requesting gestures directed toward humans since these gestures emerge frequently in apes [3] but rarely in monkeys [4], [5]; monkeys, however, can be readily trained to perform begging or pointing gestures (e.g. squirrel monkeys [6], rhesus monkeys [7] and capuchin monkeys [8]). Both begging and pointing gestures of nonhuman primates are visually-based imperative gestures produced toward a desired object (usually a food item). Begging gestures are defined as gestures produced toward an item placed just in front of a recipient or held directly in the recipient’s hand(s), whereas pointing gestures are produced toward an external item of the environment.

Requesting gestures of nonhuman primates are motorically ineffective as the emitter of the gesture extends an arm without reaching the desired object [9]. However, such a schematization of action (i.e. ritualization) does not necessarily mean that nonhuman primates understand that their gestures indicate to humans what they want. Communication between humans and nonhuman primates may occur only because the human recipient is adept at interpreting the gesture. Indeed, according to Gómez [10], nonhuman primates may produce arm extensions towards a desired object simply because they learn that these result in a human giving them the desired item; nonhuman primates would thus use requesting gestures as a conditioned response. Alternatively, nonhuman primates may comprehend the function of their arm extensions in directing the attention of humans to themselves; nonhuman primates would then use requesting gestures as intentional communication.

According to Woodruff & Premack [11], intentional communication requires that an individual not only recognizes that his behavior is informative, but also understands that another individual will perceive the informative nature of this behavior. As a consequence, Call & Tomasello [12] argued that if we are to determine how nonhuman primates comprehend their requesting gestures – whether they are a conditioned response dependent on concrete reinforcements or whether they are intentional communication – we must ask if these gestures are used flexibly and in appropriate circumstances. Several criteria can be employed to assess communication intentionality: response waiting [9], adjustments to audience, or means-ends dissociation between gestures and the context of usage [13]. Interestingly, the emitter’s adjustment to the attentional state of the recipient is the prevalent criterion in gesture studies. The emitter’s adjustment to the attentional state of the recipient is indeed required for effective and interactive communication to take place: the recipient must perceive the gestures to be able to respond to them. Hence, in the particular case of requesting gestures produced toward humans, nonhuman primates necessarily have to understand that, if their requesting gestures are to be efficient, human recipients must see these visually-based gestures.

Flexibility of gesture usage in response to the recipient’s attentional state was mostly studied in great ape species. On one hand, many studies established that great apes modify their visually-based gestures according to the attentional states of conspecific recipients: they do not gesture, or gesture much less frequently, when the conspecific recipients do not face them (e.g. chimpanzees [14]–[16], bonobos: [17], gorillas: [18] and orangutans [19]). On the other hand, studies of apes’ responses to variations in postural cues of human’s attentional state during cooperative requesting food paradigm produced mixed results. One study found that chimpanzees did not use any cues of human’s attention [20], whereas another one showed their sensitivity to the body orientation of the human recipient [21]. Furthermore, an ability to discriminate the orientation of both the body and the face of human recipients was recently revealed in every ape species including chimpanzees [22], [23]. Moreover, apes’ ability to use gaze cues of human’s attention is a much more debated issue. To date, only two studies reported chimpanzees to be able to discriminate attentive from inattentive human recipients on the basis of eyes state (eyes open *versus* eyes closed [24]; eyes looking at the monkey *versus* eyes looking at the ceiling [25]).

Only two studies have so far examined monkeys’ ability to modulate their production of requesting gestures as a function of human’s attention. They investigated the flexibility of requesting gestures usage in two species of new-world monkeys, the capuchin monkey [26] and the squirrel monkey [27]: By comparing the production of requesting gestures in a begging or a pointing situation, they found a decreased number of gestures when facing an inattentive human recipient in the begging paradigm only. Noteworthy, Hattori et al. [26] pointed that chimpanzees also failed to modify their gestures in response to human’s attentional states [20], or needed extensive training to succeed [21], in experimental designs where pointing instead of begging was elicited. Moreover, Anderson et al. [27] claimed that pointing paradigms may be more difficult to deal with than begging paradigms for nonhuman primates because, while pointing, emitters have to direct the human recipients’ attention to themselves and also to the location of food, whereas, while begging, they only have to direct the human recipients’ attention to themselves.

The present study asked whether an old-world monkey, previously trained to request food in a pointing paradigm (unpublished data), would use requesting gestures flexibly in a begging paradigm to communicate its intention to get food from a human experimenter. Specifically, we asked whether red-capped mangabeys would vary their use of begging gestures in response to variations in postural and gaze cues of human’s attention. Mangabeys are of particular interest since they already displayed their abilities to monitor the visual orientation of conspecifics as suggested in reports of social monitoring [28] and tactical deception [29]. If managabeys do understand the importance of gesturing in front of an attentive human recipient, they should be expected to emit fewer gestures and to exhibit longer latencies to start gesturing when facing an inattentive experimenter than an attentive one.

## Methods

### Ethics Statement

Experiments complied with the current French laws related to animal experimentation and were in accordance to the European directive 86/609/CEE. Animal facilities and animal care procedures are regularly monitored by the responsible local authorities. Animal husbandry and care were under management of the staff of the biological station in Paimpont, University of Rennes 1, France. On a daily routine, climbing furniture, ground substrates (woodchips and straw) and sunflowers seeds were provided as enrichment. During the experiments, animals were constantly monitored for signs of distress and care was taken to provide a stress-free experimental environment. This experiment only included behavioral observations, routine training and non-invasive contacts with the monkeys (giving food rewards) which did not require the approval of an ethics committee. The person in supplementary movie is myself, Audrey Maille, an author on this manuscript. I give permission for this to be included and published.

### Subjects

Nine red-capped mangabeys (*Cercocebus torquatus torquatus*) participated in this experiment: three subadults, 2 males and 1 female, ranging from 2 to 4 years old; and 6 adults, 1 male and 5 females ranging from 5 to 23 years old (Table 1). All subjects were housed at the Biological station (Paimpont, University of Rennes 1). They lived in a social group with access to indoor and outdoor enclosures. They were fed according to their normal daily routine, that is twice a day (fresh fruits and vegetables in the morning, monkey chows in the afternoon), and water was available *ad libitum*.

**Table 1:** Number of begging gestures and descriptive statistics for each subject over all experimental trials.

			Eyes Open				Eyes Distracted				Eyes Closed				Head Away				Body Away					
Subject	Sex	Age	N	mean	med	var	N	mean	med	var	N	mean	med	var	N	mean	med	var	N	mean	med	var		
Lorette	f	2	18	1.50	1.50	1.00	25	2.08	2.00	0.81	18	1.50	1.00	1.55	15	1.25	1.00	1.66	18	1.50	1.50	1.00		
Carillon	m	4	23	1.92	2.00	1.54	23	1.92	2.00	1.90	27	2.25	2.00	1.11	20	1.67	1.50	1.52	21	1.75	2.00	1.11		
George	m	4	44	3.67	4.00	2.79	54	4.50	5.00	2.27	49	4.08	4.00	4.99	43	3.58	2.50	1.72	39	3.25	3.00	3.84		
Chipse	f	5	23	1.92	2.00	1.54	23	1.92	2.00	1.90	27	2.25	2.00	1.11	20	1.67	1.50	1.52	21	1.75	2.00	1.11		
Julie	f	6	21	1.75	1.50	1.11	18	1.50	1.50	0.64	19	1.58	1.50	0.81	10	0.83	1.00	0.52	6	0.50	0.00	1.00		
Goffrette	f	14	18	1.50	1.00	2.27	23	1.92	1.50	4.63	10	0.83	0.00	1.61	16	1.33	0.50	2.61	13	1.08	0.50	2.45		
Pirate	m	18	25	2.08	2.00	0.99	23	1.92	2.00	1.54	28	2.33	3.00	1.33	21	1.75	2.00	0.93	17	1.42	1.50	1.17		
Chipie	f	18	12	1.00	1.00	1.27	8	0.67	0.50	0.79	8	0.67	0.50	0.61	6	0.50	0.00	0.45	2	0.17	0.00	0.15		
Zunie	f	23	11	0.92	1.00	1.36	9	0.75	0.50	0.75	20	1.67	1.00	2.61	8	0.67	0.00	1.15	2	0.17	0.00	0.15		
**Global**			–	**1.81**	**2.00**	**2.01**	**–**	**1.93**	**2.00**	**2.59**	**–**	**1.87**	**2.00**	**2.62**	**–**	**1.45**	**1.00**	**2.03**	**–**	**1.24**	**1.00**	**2.05**		

Subject: subject’s name; Sex: subject’s sex, f =  female, m =  male; Age: subject’s age in years; N: total number of begging gestures; mean; average number of begging gestures per trial; median: median number of begging gestures per trial; var: variance of the number of begging gestures per trial. See methods section for the description of the Eyes Open, Eyes Distracted, Eyes Closed, Head Away, and Body Away conditions.

The experiment was conducted in the monkeys’ home cage, which was 26.10 m^2^×3.70 m consisting of three separable units of the same size. Subjects were not deprived of food or water during testing. They were tested between 2:00 pm to 5:00 pm. The monkey being tested was isolated from dominant conspecifics, and could move freely during the test. All of the subjects had previously participated in other studies so they were all habituated to be tested and isolated.

### Procedure

Subjects received no special training prior to this experiment since all of them had learned in a previous study (conducted by AM) to produce pointing gestures to inform an experimenter about the location of one baited container among 5 available containers (unpublished data).

The experimenter (LE) squatted on the floor facing the subjects; the subjects and the experimenter being separated by a cage mesh. The experimenter held a raisin (familiar and appetent food) into her joint hands, in front of the subject’s chest. The distance (D) between the experimenter’s hands and the cage mesh depended on the subjects’ size (D: subadults  = 30 cm, adult females  = 40 cm, adult males  = 50 cm), so that the raisin was 5 cm away from the subject’s hand(s) when the subject fully extended its arm(s) through the cage mesh. Subjects were not able to touch the experimenter, since they could not reach the experimenter’s hands and limbs.

**Figure 1:**
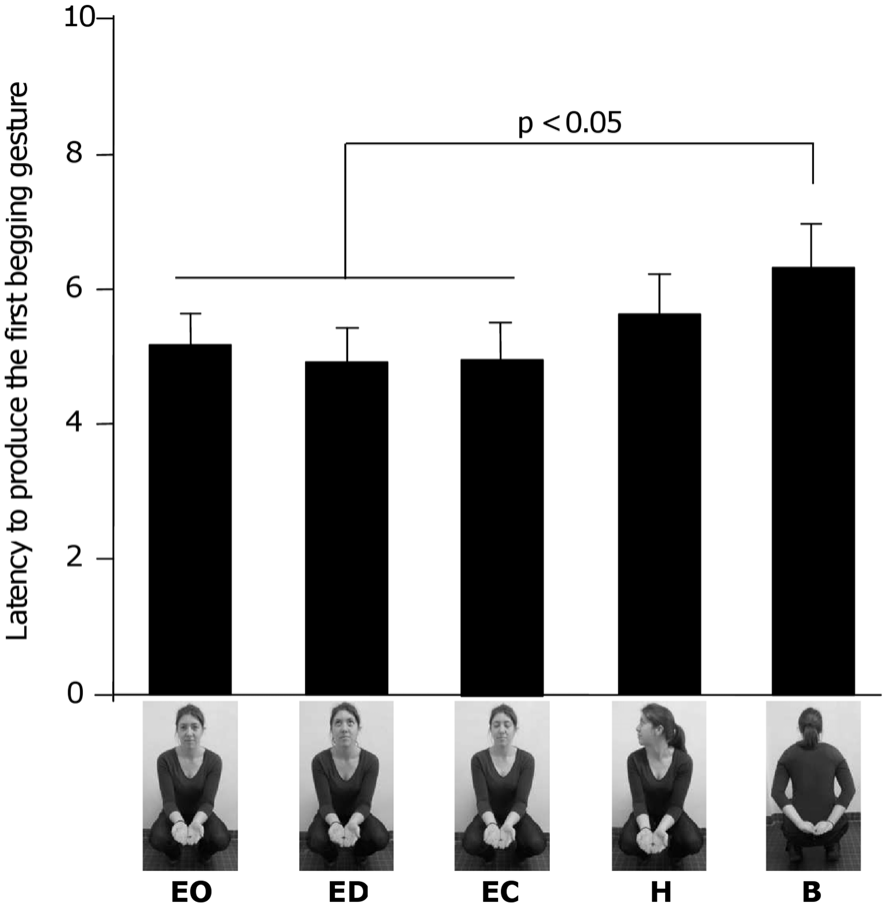
Latency to produce begging gestures in the five experimental conditions. Presented values are the means ± SE (in seconds) for the two session blocks (12 trials per experimental condition). Experimental conditions: EO  =  Eyes Open, ED  =  Eyes Distracted, EC  =  Eyes Closed, H  =  Head Away, B  =  Body Away. p<0.05: result of pairwise-*t-*test.

Two types of trials were presented alternately: motivational trials and experimental trials. In experimental trials, the experimenter held the raisin in her joint hands and engaged in one of the five following experimental conditions (Figure 1, Video S1):

1. Eyes Open: Experimenter squatted with her body and head facing the subject and with her eyes fully open and looking at the monkey’s face.
2. Eyes Distracted: Experimenter squatted with her body and head facing the subject and with her eyes fully open but looking at the ceiling.
3. Eyes Closed: Experimenter squatted with her body and head facing the subject and with her eyes closed.
4. Head Away: Experimenter squatted with her body facing the subject and with her head turned 90° away from the subject (to the left or the right side with randomization of the side).
5. Body Away: Experimenter squatted with her body and head turned 180° away from the subject and held her hands in the back.

After 10 s had elapsed (time controlled by a beeper stopwatch), the experimenter offered the subject the raisin, without regard to the subject’s behavior; and started preparing for the next trial.

In the motivational trials, the experimenter adopted the same posture than in the Eyes open condition, but she offered the raisin to the subject as soon as it produced a begging gesture.

Each session consisted of a total of 15 trials presented in a random order: 5 motivation trials and 10 experimental trials (2 repetitions of each experimental condition). Each subject participated in a total of 6 sessions, with only one session per day, thus giving a total of 12 trials per experimental condition (6 sessions × 2 repetitions of each experimental condition) for each subject.

### Data Scoring and Analysis

**Figure 2:**
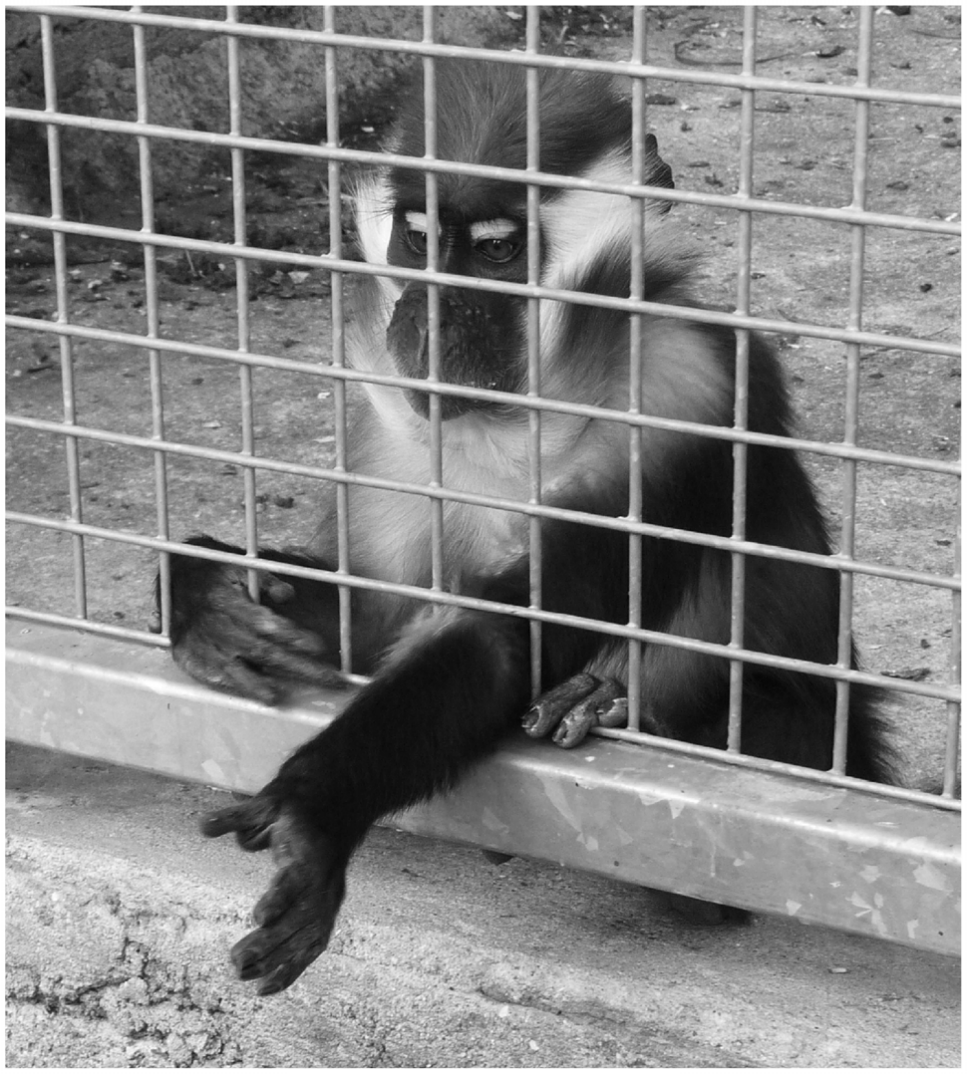
Left-arm begging gesture performed by George, a juvenile male red-capped mangabey. The arm is extended toward a raisin held in the experimenter’s hands just out of camera range. The gesture began as soon as the wrist crossed the cage mesh.

All sessions were videotaped (Sony HDD – DCR-SR58E) and later coded by the experimenter (LE). The video records were analyzed at 25 frames/s using Windows Movie Maker. A begging gesture was scored whenever the subjects extended one arm (unimanual begging) or both arms (bimanual begging) through the cage mesh (Figure 2, Video S1). A begging gesture started when the wrist(s) crossed the mesh and ended with the withdrawal of the arm(s). For a new occurrence of begging gesture to be coded, the subjects were not required to fully retract the arm(s) inside the cage but simply to bring the arm(s) back and then forth again. For each trial, the following variables were scored: 1) the latency to perform the first begging gesture (a maximum latency of 10 s. was attributed when no begging gesture was produced), and 2) the number of begging gestures. To control for a possible habituation to the experimental procedure, we pooled the sessions in two separate blocks: the first block was composed of the first 3 sessions and the second block of the last 3 sessions (i.e. 6 trials per experimental condition in each block). We assessed the effect of experimental conditions using a one-way repeated measures ANOVA (five experimental conditions), and effect of both the experimental conditions and the blocks using a two-way repeated measures ANOVA (five experimental conditions, 2 blocks) with the latency to perform the first begging gesture and the number of begging gestures as dependant variables. Pairwise-*t*-tests were used as post-hoc tests to determine which experimental conditions differ significantly from each other. All analyses were performed with R 2.10.1 and type I error α was set at 0.05.

## Results

Subjects needed on average 6.10 seconds (standard error (SE)  = 1.17) to produce the first begging gesture during experimental trials of the first block, and 4.35 seconds (SE  = 1.09) during experimental trials of the second block. The mean number of begging gestures produced by the subjects was 1.29 (SE  = 0.49) during experimental trials of the first block, and 2.03 (SE  = 0.50) during experimental trials of the second block. The analyses of both the latencies to produce the first begging gesture and the number of begging gestures produced detected a significant main effect of the blocks: subjects gestured faster (F(1,8) = 46.37, p<0.001) and more (F(1,8) = 52.77, p<0.001) in the second than in the first block. However, this habituation effect occurred similarly in all the experimental conditions since there was no interaction effect between experimental conditions and blocks both for the latency to produce the first begging gesture (F(4,32) = 1.21, p = 0.307) and for the number of begging gestures (F(4,32) = 1.01, p = 0.401).

Over all experimental trials (both blocks lumped together), subjects needed on average 4.98 seconds (SE = 1.04) to produce the first begging gesture in the Eyes Open condition, 4.75 seconds (SE = 1.08) in the Eyes Distracted condition, 4.78 seconds (SE = 1.13) in the Eyes Closed condition, 5.45 seconds (SE = 1.22) in the Head Away condition, and 6.16 seconds (SE = 1.30) in the Body Away condition. The analysis of the latency to produce the first begging gesture revealed a significant effect of the experimental condition (F(4,32) = 3.82, p = 0.005): subjects were significantly slower to gesture in the Body Away condition than in the Eyes Open (p = 0.013), Eyes Distracted (p = 0.003) and Eyes Closed condition (p = 0.004) (Figure 1). Over all experimental trials, the mean number of begging gestures produced by subjects was 1.81 (SE  = 0.47) in the Eyes Open condition, 1.93 (SE  = 0.54) in the Eyes Distracted condition, 1.87 (SE  = 0.54) in the Eyes Closed condition, 1.45 (SE  = 0.47) in the Head Away condition, and 1.24 (SE  = 0.48) in the Body Away condition (Table 1). The analysis of the number of begging gestures detected a significant effect of experimental condition (F(4,32) = 6.96, p<0.001). Subjects produced significantly less begging gestures in the Body Away condition than in the Eyes Open (p = 0.006), Eyes Distracted (p = 0.001) and Eyes Closed condition (p = 0.002). Moreover, subjects produced less begging gestures in the Head Away condition than in the Eyes Distracted (p = 0.021) and Eyes Closed condition (p = 0.004). There was no significant difference between the other experimental conditions.

**Figure 3:**
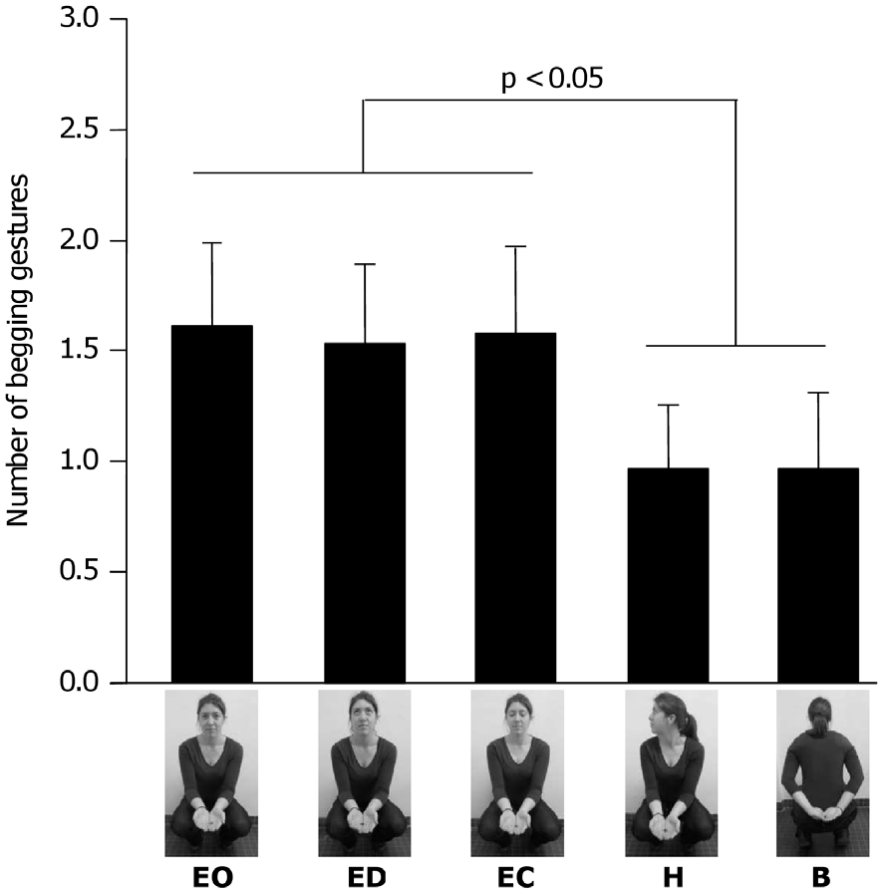
Number of begging gestures produced in the five experimental conditions. Presented values are the means ± SE for the first session block (6 first trials per condition). Experimental conditions: EO  =  Eyes Open, ED  =  Eyes Distracted, EC  =  Eyes Closed, H  =  Head Away, B  =  Body Away. p<0.05: result of pairwise-*t-*test.

We then restricted the analyses to the first block to remove the habituation effect. During experimental trials of the first block, subjects needed on average 5.53 seconds (SE  = 1.07) to produce the first begging gesture in the Eyes Open condition, 5.45 seconds (SE  = 1.08) in the Eyes Distracted condition, 5.72 seconds (SE  = 1.14) in the Eyes Closed condition, 6.83 seconds (SE  = 1.13) in the Head Away condition, and 6.97 seconds (SE  = 1.34) in the Body Away condition. The analysis of the latency to produce the first begging during the first block detected a significant main effect of the experimental condition (F(4,32) = 3.82, p = 0.005). Subjects were significantly slower to gesture in the Body Away condition than in the Eyes Open (p = 0.033) and Eyes Distracted condition (p = 0.023), and in the Head Away condition than in the Eyes Distracted condition (p = 0.041). There was no significant difference between the other experimental conditions although two other comparisons almost reached significance: subjects tended to be slower to gesture in the Body Away than in the Eyes Closed condition (p = 0.063) and in the Head Away than in the Eyes Open condition (p = 0.054). During experimental trials of the first block, the mean number of begging gestures produced by the subjects was 1.57 (SE  = 0.50) in the Eyes Open condition, 1.50 (SE  = 0.48) in the Eyes Distracted condition, 1.54 (SE  = 0.54) in the Eyes Closed condition, 0.93 (SE  = 0.40) in the Head Away condition, and 0.93 (SE  = 0.48) in the Body Away condition (Table 2). The analysis of the number of gestures produced during the first block also revealed a significant main effect of the experimental condition (F(4,32) = 6.19, p<0.001). Subjects produced less begging gestures in the Body Away condition than in the Eyes Open (p = 0.021), Eyes Distracted (p = 0.040) and Eyes Closed condition (p = 0.029). Moreover, subjects produced less begging gestures in the Head Away condition than in the Eyes Open (p = 0.021), Eyes Distracted (p = 0.040) and Eyes Closed condition (p = 0.029) (Figure 3).

**Table 2:** Number of begging gestures and descriptive statistics for each subject during experimental trials of each block.

		Eyes Open				Eyes Distracted				Eyes Closed				Head Away				Body Away			
Subject	Block	N	mean	med	var	N	mean	med	var	N	mean	med	var	N	mean	med	Var	N	mean	med	var
Lorette	1	7	1.17	1.00	0.57	9	1.50	1.50	0.30	5	0.83	0.50	1.37	2	0.33	0.00	0.27	5	0.83	1.00	0.57
	2	11	1.83	2.00	1.37	16	2.67	2.50	0.67	13	2.17	2.50	0.97	13	2.17	2.00	1.37	13	2.17	2.00	0.57
Carillon	1	6	1.00	1.00	0.80	8	1.33	1.00	0.27	8	1.33	1.00	1.87	4	0.67	1.00	0.27	7	1.17	1.00	1.37
	2	17	2.83	3.00	0.57	17	2.83	2.50	0.97	15	2.50	3.00	1.90	14	2.33	2.00	2.27	9	1.50	1.50	1.10
George	1	27	4.50	4.00	1.50	25	4.17	4.00	0.57	22	3.67	3.00	6.27	19	3.17	3.50	2.17	19	3.17	2.50	5.80
	2	17	2.83	3.00	2.97	29	4.83	5.00	4.17	27	4.50	4.50	4.30	24	4.00	4.00	1.20	20	3.33	3.50	2.67
Chipse	1	14	2.33	2.50	1.87	13	2.17	2.50	3.37	15	2.50	2.50	1.10	8	1.33	1.00	1.07	11	1.83	2.00	1.77
	2	9	1.50	1.50	1.10	10	1.67	1.50	0.67	12	2.00	2.00	1.20	12	2.00	2.00	2.00	10	1.67	1.50	0.67
Julie	1	10	1.67	1.50	0.67	8	1.33	1.00	0.27	10	1.67	1.50	0.67	5	0.83	1.00	0.57	2	0.33	0.00	0.67
	2	11	1.83	2.00	1.77	10	1.67	2.00	1.07	9	1.50	1.50	1.10	5	0.83	1.00	0.57	4	0.67	0.00	1.47
Goffrette	1	2	0.33	0.00	0.27	1	0.17	0.00	0.17	0	0.00	0.00	0.00	0	0.00	0.00	0.00	0	0.00	0.00	0.00
	2	16	2.67	2.50	1.47	22	3.67	3.50	2.67	10	1.67	1.50	1.87	16	2.67	2.50	1.47	13	2.17	1.50	2.57
Pirate	1	11	1.83	2.00	1.37	12	2.00	2.00	2.00	16	2.67	3.00	1.07	9	1.50	1.50	1.10	6	1.00	1.00	0.80
	2	14	2.33	2.50	0.67	11	1.83	2.00	1.37	12	2.00	2.50	1.60	12	2.00	2.00	0.80	11	1.83	2.00	1.37
Chipie	1	4	0.67	0.00	1.47	2	0.33	0.00	0.27	3	0.50	0.00	0.70	2	0.33	0.00	0.27	0	0.00	0.00	0.00
	2	8	1.33	1.00	1.07	6	1.00	1.00	1.20	5	0.83	1.00	0.57	4	0.67	0.50	0.67	2	0.33	0.00	0.27
Zunie	1	4	0.67	1.00	0.27	3	0.50	0.00	0.70	4	0.67	0.50	0.67	1	0.17	0.00	0.17	0	0.00	0.00	0.00
	2	7	1.17	0.50	2.57	6	1.00	1.00	0.80	16	2.67	2.50	2.67	7	1.17	1.00	1.77	2	0.33	0.00	0.27
**Global**	**1**	–	**1.57**	**1.00**	**2.29**	**–**	**1.50**	**1.00**	**2.10**	**–**	**1.54**	**1.00**	**2.59**	**–**	**0.93**	**1.00**	**1.43**	**–**	**0.93**	**0.00**	**2.03**
	**2**	–	**2.04**	**2.00**	**1.66**	**–**	**2.35**	**2.00**	**2.76**	**–**	**2.20**	**2.00**	**2.47**	**–**	**1.98**	**2.00**	**2.09**	**–**	**1.56**	**1.00**	**1.91**

Subject: subject’s name; Block: block of 6 trials per condition, 1 =  first block, 2 =  second block; N: total number of begging gestures; mean: average number of begging gestures per trial; median: median number of begging gestures per trial; var: variance of the number of begging gestures per trial. See methods section for the description of the Eyes Open, Eyes Distracted, Eyes Closed, Head Away, and Body Away conditions.

## Discussion

The results of this study indicate that mangabeys modified their production of begging gestures, in response to some postural cues of human’s attentional state. Mostly, mangabeys were sensitive to the front-back body orientation of the experimenter and gestured more and faster when the body of the experimenter was oriented toward them as opposed to away from them. They were also sensitive to the experimenter’s head orientation: they begged faster (at least during the first trials) and more often when the head of the experimenter was oriented toward them as opposed to turned away from them. In contrast, they did not respond to subtle changes in the experimenter’s eyes state since they gestured at the same level whether the eyes of the experimenter were open or closed, averted toward them or upon them. In sum, mangabeys responded preferentially to a human facing them regardless of her eyes state. This is the first demonstration, to our knowledge, of such a flexible use of an acquired requesting gesture by an old-world monkey.

One could argue that the fact that mangabeys begged for food in all experimental conditions suggests that they partly reacted to the sight of the desired food. The raisin was, indeed, visible to mangabeys in all experimental conditions, even in the Body Away condition (i.e. the experimenter held the raisin in her back in this experimental condition). However, mangabeys gestured significantly less often when the experimenter was not facing them, which indicates that their begging gestures are more than just a conditioned response to the mere presence of desired food. Moreover, mangabeys did not only respond to the experimenter’s general capacity to offer food since they lowered their gestural behavior in the Head Away condition though the experimenter was in an appropriate posture to deliver the food (i.e. frontal body orientation). Noteworthy, like chimpanzees [22], mangabeys responded to the head orientation from the beginning of testing, without preparatory training and further differential reinforcement (i.e. all subjects were rewarded at the end of the trial regardless of how much they gestured). In sum, our results show that mangabeys did not merely respond to the sight of a desired food or to the experimenter’s general capacity to offer food, but rather that they gestured preferentially when the experimenter was attending to them. It thus appears that mangabeys’ begging gestures were controlled by the emitter as a function of attentional cues displayed by the human recipient. Hence, mangabeys’s arm extensions seem to represent a bid to communicate their wanting of food to a human experimenter.

Moreover, mangabeys did not only decrease the number of their begging gestures when both the body and the head of the experimenter were oriented away, but also slowed down their production of begging gestures. This result highlights mangabeys’ ability to slow down their production of requesting gestures toward an appetent food item when facing an inattentive human recipient. Only two studies measured apes’ latency to produce requesting gestures during cooperative requesting paradigm, and they showed species-specific differences: chimpanzees [30] and gorillas [31] were slower to gesture when the body of the experimenter was oriented away from them, whereas orangutans failed to alter their willingness to request food in the same condition [31]. Thus, our study reveals that, in a cooperative requesting paradigm, mangabeys were able of self-control without extensive learning, similarly to chimpanzees and gorillas, and furthermore, that they overcame orangutans. Yet, in a reverse-reward contingency task, where they had to inhibit their strong tendency to point toward the larger of two quantities of appetent food items, mangabeys (*Cercocebus torquatus lunatus*) [32] where shown to need longer training to learn to solve the task than great apes, including orangutans [33]. Altogether these findings suggest that a begging paradigm might be more appropriate than a pointing paradigm to compare the inhibitory abilities of mangabeys and ape species.

Considering that mangabeys seem to understand that facing an attentive human recipient is a prerequisite for a successful communication in the visual domain, they may be able to deploy attention-getting behaviors to manipulate the attention of human recipients. Chimpanzees were, indeed, shown to increase their usage of attention-getting behaviors when facing an inattentive recipient: they produced more vocalizations [24], auditory-based gestures [34] and tactually-based gestures [15]. In our experiment, however, mangabeys produced a low rate of vocalizations and no cage banging, clapping, throwing or patting (i.e. but remember that they were unable to touch the experimenter since they could not reach her limbs; see method). We assume that, in our experiment, mangabeys did not produce attention-getting behaviors because of the short trials’ duration (i.e. 10 s). In a future study, a longer exposure to an inattentive human experimenter may prompt mangabeys to develop new communicative strategies in case their begging gestures are ineffective.

Besides, one could ask why mangabeys did not respond to the human’s eyes state although the primate brain contains neurons selectively responsive to eye gaze [35]. One possible explanation may be that mangabeys are not predisposed to focus primarily on conspecifics’ eyes to assess their attentional state. Red-capped mangabeys are semi-arboreal and live in dense forests where the visibility is often reduced. So, relying on gaze cues to determine the direction of conspecific recipients’ attention might be irrelevant for them when the eyes of groupmates are occluded or in shadow. Moreover, according to Emery [36], the orientation of the whole head could be a sufficient indicator of attention direction since, in most cases of social interaction, there is a strong correlation between the direction of head and eyes. Another conceivable explanation may be that mangabeys’ lack of sensitivity to the eyes state is restricted to the human gaze. Indeed, for nonhuman primates, assessing humans’eyes state seems to require extensive exposure. For instance, when exposed to paired photographs, rhesus monkeys (*Macaca mulatta*) were shown to need long training (29 to 157 trials) to be able to discriminate direct gaze from distracted gaze of human models [37] whereas they spontaneously react to similar photographs of conspecifics [38]. Three no mutually exclusive hypotheses might explain such a difficulty for nonhuman primates to assess human’s eyes state. First, nonhuman primates might use subtle gaze cues to assess gaze direction of conspecifics which are reduced in the human face such as gaze timing and the highlighting coloration of their eye region (for instance in mangabeys : velocity and duration of gazes [39], contrasting colors between white eyelids and red eyebrows, Figure 2, Video S1). Second, close contact to humans might be necessary to promote understanding of human gaze as suggested by studies which revealed sensitivity to eyes state during requesting paradigm in enculturated apes (e.g. (orangutans: [12], chimpanzees: [25], and gorillas: [40]; and see also studies in domesticated mammals: dogs: [41], horses: [42], [43]). Third, looking at the eyes of a human facing them might be frightening for nonhuman primates. Likewise, gaze aversion was reported in rhesus monkey (*Macaca mulatta*) facing a human observer [44].

Interestingly, in contrast to mangabeys, capuchin monkeys [26] and chimpanzees [24] were shown to produce more begging gestures when the experimenters could see them (Eyes Open) than when they could not (Eyes Closed). Capuchin monkeys [26] and chimpanzees [24] are yet semi-arboreal species too, and they were tested in similar conditions (i.e. cooperative begging paradigm) and probably experienced as few interactions with humans (i.e. laboratory animals without enculturation) as did our mangabeys. Noteworthy, when exposed to a human face, capuchin monkeys [45] and chimpanzees [46] were shown to look at a direct gaze longer than an averted gaze. A lack of aversion to the human gaze might thus explain why, in a cooperative requesting paradigm, capuchin monkeys [26] and chimpanzees [24], unlike our mangabeys, responded to the gaze direction of the experimenter. Finally, it should be noticed that cooperative paradigm might limit the expression of monkeys’ sensitivity to the human’s eye state. Indeed, rhesus monkeys who appeared unable to use gaze direction of human individuals in a cooperative paradigm (i.e. object-choice task [47]) actually succeeded in a competitive paradigm (i.e. pilfering task [48]). Perhaps a competitive paradigm would better approximate the normal conditions in which mangabeys naturally use humans’ visual perception as a cue to assess their attentional state.

Our study showed that the production of an acquired begging gesture in an old-world monkey species, the red-capped mangabey, varied as a function of postural cues of human’s attention, which were the head and the body orientation. These results suggest that mangabeys can understand the orientation of human recipients (and thus their attention) as a prerequisite for successful communication in the visual domain. Our finding provides important evidence that captive mangabeys trained to produce requesting gestures use them in flexible way, that is to say, according to whether they will be perceived by human recipients. We propose that the flexible use of this acquired gesture represents a genuine case of gestural communication toward a heterospecific recipient. This result is an important addition to the knowledge about old-world monkeys’ ability to communicate their intentions to get some food, or more generally goods, in a cooperative context.

## Supporting Information

Video S1 Chipse, an adult female red-capped mangabey, produces requesting gestures by extending an arm through the cage mesh toward an experimenter who holds a raisin in her hands. The experimenter displays five experimental conditions in succession in which her attentional state differs on the basis of gaze (Eyes Open, Eyes Distracted, and Eyes Closed) head (Head Away) and body (Body Away) orientation.
(WMV)
